# The Role of Mucosal Immunity in the Pathogenesis of Necrotizing Enterocolitis

**DOI:** 10.3389/fped.2017.00040

**Published:** 2017-03-03

**Authors:** Zerina Hodzic, Alexa M. Bolock, Misty Good

**Affiliations:** ^1^University of Pittsburgh School of Medicine, Pittsburgh, PA, USA; ^2^Division of Newborn Medicine, Department of Pediatrics, Washington University School of Medicine, St. Louis, MO, USA

**Keywords:** necrotizing enterocolitis, innate immunity, mucosa, intestine, toll-like receptor 4, human milk oligosaccharide, microbiota, prematurity

## Abstract

Necrotizing enterocolitis (NEC) is the most devastating gastrointestinal disease of prematurity. Although the precise cause is not well understood, the main risk factors thought to contribute to NEC include prematurity, formula feeding, and bacterial colonization. Recent evidence suggests that NEC develops as a consequence of intestinal hyper-responsiveness to microbial ligands upon bacterial colonization in the preterm infant, initiating a cascade of aberrant signaling events, and a robust pro-inflammatory mucosal immune response. We now have a greater understanding of important mechanisms of disease pathogenesis, such as the role of cytokines, immunoglobulins, and immune cells in NEC. In this review, we will provide an overview of the mucosal immunity of the intestine and the relationship between components of the mucosal immune system involved in the pathogenesis of NEC, while highlighting recent advances in the field that have promise as potential therapeutic targets. First, we will describe the cellular components of the intestinal epithelium and mucosal immune system and their relationship to NEC. We will then discuss the relationship between the gut microbiota and cell signaling that underpins disease pathogenesis. We will conclude our discussion by highlighting notable therapeutic advancements in NEC that target the intestinal mucosal immunity.

## Introduction

Necrotizing enterocolitis (NEC) is notably the most lethal gastrointestinal disease of premature infants. The disease prevalence is approximately 7% of infants born between 500 and 1,500 g in the United States and Canada (1–3). Unfortunately, both the treatment approach and mortality have remained unchanged for decades with a mortality rate as high as 42% (4). At the same time, NEC represents a significant economic burden on the health system with an estimated annual cost upwards of one billion dollars in the United States (2, 5–7). These challenges fuel the need to understand disease pathogenesis so as to develop novel therapeutic and preventative strategies. Presently, it is believed that the immature intestine of premature infants exists in a hyper-active state due to abnormal bacterial colonization, which ultimately results in a robust inflammatory response and impairment of intestinal perfusion, thereby predisposing infants to NEC (2, 7, 8). Current research suggests the intestinal immune system is intricately involved in this process, which is comprised of the intestinal epithelium, immune cells, and commensal bacteria that maintain gastrointestinal homeostasis. Here, we will review the current knowledge of intestinal mucosal immunity in relation to NEC. First, we will discuss the cell types that comprise the intestinal immune system with attention to how these cells are involved in NEC. We will then describe the role of the innate immune system with specific attention to toll-like receptor 4 (TLR4) signaling in the pathogenesis of NEC. We will then review the role of gut microbiota in our current understanding of this disease. Finally, we will describe advancements in potential treatment strategies rooted in our current understanding of the relationship between mucosal immunity and the development of NEC.

## Cells of the Intestinal Immune System

In order to understand the pathogenesis of NEC, it is important to appreciate the role of the immune system in the maintenance of gastrointestinal homeostasis. We will first describe the role of epithelium and immune cells in mucosal immunity and then describe their role in the development of NEC with specific attention to the interplay of these cell types and the signaling pathways involved.

### The Intestinal Epithelium

**Figure 1:**
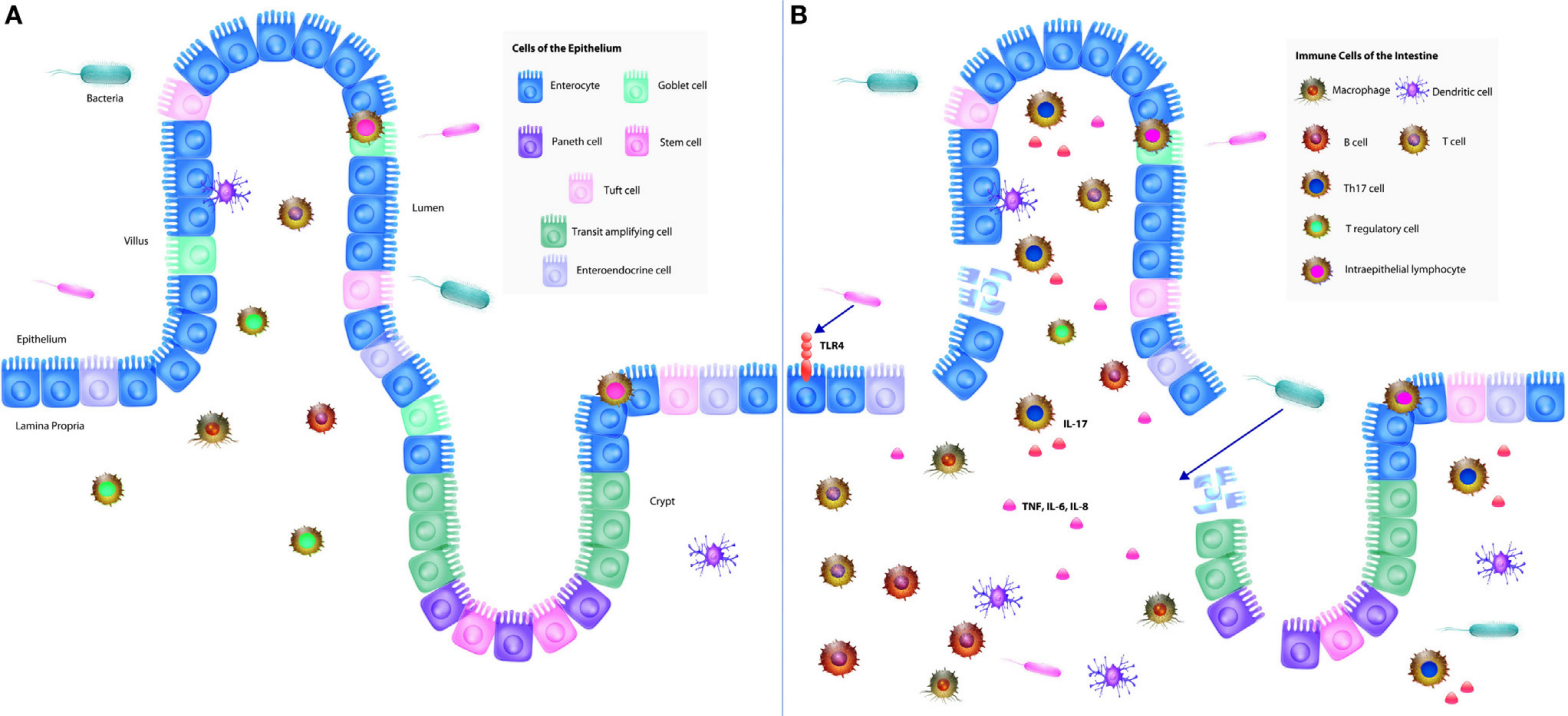
**The neonatal intestinal immune system and its interaction in the pathogenesis of necrotizing enterocolitis (NEC)**. **(A)** The intestinal mucosal immune system is comprised of the cells of the epithelium, immune cells, and commensal bacteria. The epithelium consists of villi and crypts. Enterocytes, goblet cells, enteroendocrine cells, and tuft cells exist within the villi, whereas Paneth cells and stem cells occupy the base of the crypts. Immune cells consist of intraepithelial lymphocytes, T regulatory cells (Tregs), T cells, B cells, macrophages, and dendritic cells, which reside predominantly in the lamina propria underlying the epithelium. Commensal bacteria inhabit the lumen of the gut, which constantly interact with the epithelium and immune cells to help maintain protection against pathogenic bacteria. **(B)** In NEC, lipopolysaccharide from Gram-negative bacteria interact with toll-like receptor 4 (TLR4) expressed by predominantly by enterocytes, which results in the breakdown of the gut barrier, allowing for pathogenic bacterial translocation. A pro-inflammatory response follows resulting in increased production of pro-inflammatory cytokines (IL-6, IL-8, and TNF) as well as increased Th17 cells and decreased Tregs. The combination of these cellular responses with TLR4 signaling results in a profound inflammatory response and subsequently NEC.

The epithelium represents the first layer of defense, comprised of at least seven differentiated cell types that together maintain barrier integrity and provide defense against pathogens with the presence of tight junctions (9). The epithelium has two distinct structures: the villus and the crypt. The villus contains enterocytes, goblet cells, enteroendocrine cells, and tuft cells, whereas the crypt houses transit amplifying cells, Paneth cells, and stem cells (Figure 1) (10). Stem cells expressing leucine-rich containing G protein-coupled receptor 5 (Lgr5) are capable of generating all cell types of the epithelium (11, 12). Together, these cells comprise the epithelium, which we will now discuss in further detail.

### Enterocytes

Enterocytes (IECs) are the predominant absorptive cells of the epithelium defined by the presence of microvilli, but the role of enterocytes is not limited to nutrient absorption; rather, they are important cells in the preservation of intestinal integrity and mucosal immunity (13–16). IECs, as the most numerous cells of the epithelium, provide the physical barrier between the lumen of the gastrointestinal tract and the lamina propria *via* the maintenance of tight junctions (14). They originate from within the intestinal crypts and migrate along the villi, at which point they undergo apoptosis, renewing the epithelium every 3–5 days in a continuous cycle of IEC proliferation, migration, and apoptosis in mouse studies (17). This cycle is crucial for intestinal homeostasis; however, aberrancy in this process can lead to disastrous effects, such as bacterial translocation, which we will discuss in the context of NEC in a later section (18). IECs are notable for the presence of several pattern-recognition receptors (PRRs), such as toll-like receptors (TLRs) and nucleotide-binding oligomerization domains (NODs), thereby aiding in the clearance of pathogenic bacteria while maintaining a population of commensal bacteria (15, 16, 19, 20). We will describe TLRs and NODs more extensively in a later section with specific attention to the relationship between TLR4 expression by enterocytes and gut barrier integrity. Moreover, IECs also express major histocompatibility class (MHC) I and II molecules and non-classical MHC molecules, allowing IECs to process and present antigens to the immune cells of the intestine (21, 22). In doing so, there is a direct communication between the antigens within the lumen and the cells of the lamina propria (Figure 1). Accordingly, enterocytes are vital cells of the epithelium with the roles in the maintenance of the gut barrier and commensal bacteria, absorption of nutrients, and communication with the immune cells of the lamina propria.

### Goblet Cells

Goblet cells are particularly important with their role in generating the mucus layer of the intestine, preventing the interaction between pathogenic bacteria and the epithelium, while providing support for commensal bacteria, antimicrobial peptides (AMPs), and secretory immunoglobulin A (IgA) (23). Moreover, goblet cells are also capable of delivering luminal antigens to a subset of underlying dendritic cells (DCs), CD103^+^ lamina propria DCs, which have tolerogenic properties, thereby assisting with the maintenance of commensal bacteria and intestinal homeostasis (24).

Goblet cell differentiation is determined by the activity of the Notch signaling pathway (25). Sodhi et al. (26) determined that the innate immune receptor TLR4 regulates Notch signaling and subsequent goblet cell development in the small intestine, such that TLR4 signaling prevented goblet cell differentiation independent from the influence of the microbiota. Furthermore, Notch signaling was found to be increased in mice as well as premature infants with NEC, whereas inhibition of the Notch pathway led to an increased number of goblet cells and attenuated experimental NEC in mice (26). This study highlights the regulation by TLR4 and Notch signaling in NEC pathogenesis.

Goblet cells secrete glycoproteins called mucins, of which, the Muc2 mucin is of critical importance in maintaining an inner mucus layer impervious to pathogenic bacteria, while simultaneously creating an outer mucus layer and providing an ideal habitat for commensal bacteria (27). Notably, ileal Muc2 is decreased in NEC and depletion of intestinal goblet cells increases susceptibility and severity of experimental NEC (28, 29). This subsequent decrease in mechanical defenses increases the vulnerability of the epithelium to pathogenic bacteria (26, 30), which can be further exacerbated by decreased intestinal motility in the setting of prematurity (31, 32). Abnormal goblet cell function is implicated in the development of NEC and mechanisms to enhance goblet cell production and/or function may provide a unique way to prevent the disease.

### Paneth Cells

Paneth cells also provide a unique source of protection in the maintenance of the intestinal barrier. Paneth cells produce AMPs, lysozyme, secretory phospholipase A2, C-lectin RegIIIγ, α- and β-defensins, and angiogenin-4 to protect the host from pathogenic bacteria while shaping the composition of the microbiota (33, 34). Paneth cells and their AMPs, particularly α-defensins, have been implicated in diseases of the intestine through the use of several animal models (33). One noteworthy study utilized two genetic mouse models to study the role of α-defensins, including *DEFA5*-expressing transgenic mice and also mice that are deficient in matrix metalloproteinase 7 (*MMP7*), which is required to activate α-defensins (35). Using 16S rRNA sequencing, they found there was an α-defensin-dependent change in the composition of the microbiota, such that there was a correlation between α-defensin deficiency, decreased populations of bacteria from the phylum Bacteroidetes, and increased populations of bacteria from the phylum Firmicutes (35). Moreover, in *DEFA5* transgenic mice, there was a loss of segmented filamentous bacteria and interleukin 17 (IL-17)-producing lamina propria T cells, substantiating the role of α-defensins and thus, Paneth cells in influencing the microbiota of the gut and modulating the intestinal immunologic response to pathogens (35).

The role of Paneth cells in disease has been well characterized in the studies on inflammatory bowel disease (IBD), specifically ileal Crohn’s disease (36). There are several susceptibility genes associated with Crohn’s disease, such as nucleotide-binding oligomerization domain-containing protein 2 (*NOD2*), which is expressed predominantly by Paneth cells in the small intestine (37–39). One such study evaluated known *NOD2* loss-of-function gene mutations known to be risk factors for IBD in a group of very low birth weight infants (40). They found that the presence of two or more *NOD2* genetic risk factors was an independently associated with the development of NEC and focal intestinal perforation (40). Moreover, *NOD2*-deficient mice develop ileal granulomatous inflammation in response to inoculation with *Helicobacter hepaticus* characterized by increased inflammatory cytokines and expression of Th1-related genes (41). This was restored with transgenic expression of α-defensin in Paneth cells (41). Moreover, in ileal Crohn’s disease, there is decreased expression of α-defensins, suggesting a relationship between the secretion of α-defensins by Paneth cells and the pathogenesis of Crohn’s disease. The involvement of Paneth cells in mediating protection against NEC is not well defined. However, animal models utilizing a Paneth-cell depletion method in the presence of *Klebsiella pneumoniae* have been shown to produce NEC-like intestinal injury, suggesting Paneth cells may have a role in NEC pathogenesis (42–44).

### Enteroendocrine Cells

Enteroendocrine cells encompass several cell types that are located throughout the gastrointestinal system and primarily act to secrete hormones in response to food stimuli (45). The role of enteroendocrine cells in the promotion of mucosal immunity is incompletely understood; however, there is interplay between the immune system and enteroendocrine cells (46). For example, enteroendocrine cells express TLRs, and accordingly, can release chemokines and defensins in response to bacterial antigens, suggesting these cells have a role in the maintenance of intestinal homeostasis with respect to bacterial colonization (47, 48). Interaction with the gut microbiota is not limited to the expression of TLRs by enteroendocrine cells. Rather, there are several mechanisms by which these cells cooperate with the microbiota (49). For example, L cells are a subset of enteroendocrine cells that secrete glucagon-like peptides 1 and 2 (GLP-1 and GLP-2) (45). Short-chain fatty acids (SCFAs) derived from gut microbiota can activate specific receptors of L cells, thereby influencing energy metabolism and gut barrier function *via* secretion of GLP-1 and GLP-2 (49). Accordingly, enteroendocrine cells have a unique interaction with the gut microbiota.

Furthermore, enteroendocrine cells have also been implicated in IBD. Friedrich et al. (50) found that patients have elevated serum and colonic pro-inflammatory IL-17c and discovered that enteroendocrine and to a lesser extent goblet cells were the main producers of IL-17c. This demonstrates a relationship between the neuroendocrine system of the gut and the Th17 pathway of the immune system (50). Nonetheless, more studies are necessary to understand the role of enteroendocrine cells during intestinal inflammation and more specifically NEC.

### Tuft Cells

Finally, there is a unique cell type of the intestinal epithelium called tuft cells, which are thought to be taste-chemosensory cells found in both the respiratory tract and gastrointestinal tract (51, 52). Tuft cells were initially identified by electron microscopy due to their unique morphology with their tubulovesicular system and a tuft of long, blunt microvilli (51, 52). There is increasing evidence for tuft cell involvement in immunity (53). More specifically, there is a newly discovered role of tuft cells in parasitic infections, whereby, they generate type 2 helper T cell responses to promote immunity against such infections (54–56). This is accomplished by the ability to secrete IL-25 in a transient receptor potential cation channel subfamily M member 5 (Trpm5)-dependent mechanism (51, 54–56). IL-25 promotes the expansion of type 2 innate lymphoid cells, an important source of IL-13 (56). This is crucial because IL-13 acts to promote differentiation of both tuft and goblet cells contributing to the “weep and sweep” response of the intestine to parasitic helminth infections, which consists of goblet cell hyperplasia and increased smooth muscle contractility (56). Although there is currently no known role of tuft cells in the pathogenesis of NEC, these studies provide evidence that these cells are actively involved in the mucosal immunity of the intestine in addition to their chemosensory roles.

### Immune Cells

Immune cells that compose the innate and adaptive immunity of the intestine exist in the epithelium and lamina propria (57). The epithelium houses intraepithelial lymphocytes (IELs) and the cytoplasmic extensions of DCs that interdigitate between epithelial cells (23, 57). Several cell types inhabit the lamina propria, including but not limited to DCs, macrophages, neutrophils, immunocompetent T and B cells, and T regulatory cells (Tregs) in addition to mesenchymal cell types, such as endothelial cells (23, 57). We will now discuss the various immune cells implicated in NEC, including T cells, Tregs, Th17 cells, IELs, B cells, macrophages, and DCs.

### T Cells

There is increasing evidence that immune cells have a primary role in the development of NEC, including but not limited to T cells. In one study, NEC was induced in recombination activating gene 1-deficient (*Rag1^–/–^*) mice, which lack functional T and B cells (58, 59). Compared to wild-type counterparts, these animals exhibited significantly decreased intestinal injury and mucosal cytokine production, suggesting they are protected from NEC development (59). Demonstrating that these cells have a causative role in NEC pathogenesis, susceptibility to NEC was restored with the adoptive transfer of naïve CD4^+^ T cells (59). Furthermore, recruitment and differentiation of T cells were found to be mediated by activation of TLR4 *via* cognate chemokine ligand 25 (59). Thus, this study demonstrates the principal role of immune cells in NEC, specifically CD4^+^ T cells, and their relationship to TLR4 signaling.

### Relationship between Tregs and Th17 Cells

The roles of immune cells in NEC are dynamic, and there is an imbalance of immune cells that favor a exaggerated pro-inflammatory state. This is remarkably demonstrated by the relationship between Tregs and Th17 cells. Forkhead box P3 (Foxp3)-expressing Treg cells are essential for the maintenance of a stable equilibrium in the gut (57, 60). Tregs are important in counterbalancing inflammation and promoting antigen-specific IgA responses so as to maintain commensalism with gut microbiota (57, 61). In humans, intestinal Tregs are present as early as 23 weeks of gestation, suggesting an early role in intestinal homeostasis (60). In the intestinal lamina propria of both mice and premature infants with NEC, there is decreased production of Tregs and increased production of CD4^+^ Th17 cells mediated by STAT3 (59). Additionally, expression of the IL-17 receptor, IL-17RA is significantly increased in a TLR4-mediated manner (59). Importantly, IL-17 release by CD4^+^ Th17 cells results in intestinal mucosal injury, as demonstrated by impaired enterocyte tight junctions, increased enterocyte apoptosis, and decreased enterocyte proliferation, all of which are hallmark features of NEC (59). Similarly, in a study analyzing the T cell populations in human NEC, there was decreased Foxp3^+^ Tregs relative to effector T cells compared to controls, which correlated with a mucosal cytokine expression profile indicative of inhibited Treg expression (62). With NEC resolution, Treg cell populations recovered, suggesting either the inflammatory response observed in NEC decreases Treg populations acutely or inflammation is attenuated as Treg populations increase with mucosal healing (62).

The premature human gut is notable for increased expression of pro-inflammatory cytokines, specifically IL-6, which is involved in T cells differentiating toward a Th17 phenotype during NEC development (59). Importantly, in a mouse model of NEC, oral administration of all-trans retinoic acid (ATRA), which binds to the nuclear retinoic acid receptor to stabilize transcription of Foxp3 and repress transcription of RAR-related orphan receptor γt, resulted in decreased NEC severity, increased Tregs, decreased CD4^+^ Th17 cells, and attenuation of IL-17 expression (59). This suggests that the Tregs serve to provide a protective role in intestinal homeostasis, which is disrupted in NEC.

Further studies demonstrated that Th17 recruitment and Treg depletion resulted in apoptosis of Lgr5^+^ intestinal stem cells within the crypts of Lieberkühn in an experimental model of NEC (63). After exposure to recombinant IL-17, enteroids derived from intestinal stem cells exhibited decreased proliferation, decreased differentiation, and increased apoptosis (63). In addition, depletion of Tregs led to increased intestinal crypt apoptosis, exacerbating the experimental murine NEC model (63). Importantly, these effects were reversed upon administration of ATRA with its ability to modulate lymphocyte populations toward a protective Treg phenotype (63). This highlights the potential use of retinoic acid as a therapeutic in the treatment and prevention of NEC. Taken together, NEC is an immune cell-mediated disease defined by an imbalance of lymphocytes shifted toward a Th17 cell phenotype with a decrease in Tregs, which is destructive in this specific setting.

### Intraepithelial Lymphocytes

Intraepithelial lymphocytes are a group of heterogeneous lymphocytes of the innate immune system in the distinctive position to interact dynamically with the local gut environment and the mucosal adaptive immune system to maintain intestinal homeostasis (64). This unique population of IELs bears the T cell receptor γδ (65). They differ from TCRαβ T-cells in that they do not require antigen processing for effector function (65). They are important in the discussion of the infant gut due to their early development in embryogenesis (66, 67). IELs are involved in a multifaceted approach in the maintenance and repair of the epithelium through tight junction preservation, recognition of stressors, and regulation of inflammation (65). They are also involved in a dynamic cross talk with commensal bacteria, as commensal bacteria direct expression of key immunomodulatory and antibacterial responses by influencing gene transcription of IELs, while IELs prevent opportunistic commensal bacteria overgrowth in the setting of mucosal injury (68). Weitkamp et al. (66) determined that γδ IELs are the predominant IEL subtype in the immature intestine of mice and premature infants. Furthermore, they showed that in the intestine of infants with NEC, there was a reduction in the γδ IEL subset as compared to non-NEC controls (66). This study highlights the important role of γδ IELs in intestinal barrier protection and suggests a potential cellular target for NEC prevention.

### B Cells and Immunoglobulins

B cells are both important antigen-presenting cells (APCs) and integral members of adaptive immunity with their ability to secrete immunoglobulins as plasma cells, most notably IgA in the context of neonatal immunity. IgA can neutralize pathogenic bacteria to maintain intestinal homeostasis (9). The maternal IgA from colostrum and milk is an important source of immunity in the neonatal period. Secretory IgA (sIgA) is detected weeks after birth and increases progressively; however, preterm infants have decreased concentrations of sIgA compared to full-term infants (69, 70). One study found higher levels of sIgA from the colostrum and milk of mothers of preterm infants, suggesting immunological adaptation occurs in the setting of prematurity to enhance immunity with IgA and reinforces the benefits of breastfeeding in this context (71). There are both early and long-term benefits of early exposure to maternal sIgA (72). In one study, they found weanling mice exposed to sIgA-deficient breast milk displayed increased colonization of draining lymph nodes with bacteria, specifically aerobic bacteria and the opportunist pathogen *Ochrobactrum anthropi* (72). At both weaning and adulthood, there were significant differences in the gut microbiota and the pattern of epithelial gene expression of the intestine, including genes associated with IBD, between these mice compared to mice exposed to sIgA (72). Accordingly, exposure to sIgA in breast milk promotes intestinal homeostasis in the neonatal period with long-term implications (72). Taken together, IgA is important in intestinal immunity, and maternal IgA is crucial given the immaturity of the immune system during the neonatal period.

### Macrophages

Macrophages are important phagocytic and bactericidal cells of the immune system. Macrophages are activated after exposure to lipopolysaccharide (LPS) and interferon-γ, which then release pro-inflammatory cytokines and nitric oxide (57). To counteract excessive inflammation, intestinal macrophages are inhibited by tumor growth factor-β (TGF-β) (57) and have been implicated in NEC pathogenesis (73). Intestinal macrophages from healthy term infants have increased phagocytic and bactericidal activity as well as minimal inflammatory cytokine production when exposed to bacterial products, which is attributed to the anti-inflammatory effects of transforming growth factor β_2_ (TGF-β_2_) (73). In contrast, the phenotype of macrophages during NEC is strongly inflammatory as demonstrated by increased expression of the gene, mothers against decapentaplegic homolog 7 (*Smad7*), an inhibitor of TGF-β_2_ (74, 75). Consequently, this interrupts TGF-β-mediated downregulation of the pro-inflammatory response by macrophages in NEC and thereby sensitizes macrophages to bacterial products, leading to a significant pro-inflammatory response (74). Taken together, the imbalance of macrophage effector responses toward an inflammatory state increases mucosal injury in NEC.

### Dendritic Cells

Dendritic cells are specialized APCs that have the delicate role of mediating protective adaptive immunity in response to pathogens while maintaining intestinal homeostasis and commensal bacteria (76). Although they are important in intestinal immunity, the role of DCs in NEC has not been fully elucidated. However, in one study using *Cronobacter sakazakii* to induce NEC in newborn mice, DCs were recruited from the lamina propria, resulting in intestinal barrier dysfunction *via* tight junction disruption and increased enterocyte apoptosis *via* TGF-β production (77). The study concluded that the presence *C. sakazakii* was able to modulate the activity of DCs to increase TGF-β production, which subsequently resulted in damage to the mucosal barrier integrity (77). This highlights the sensitive interaction between DCs and bacteria, which can be detrimental in the setting of NEC; however, more studies are necessary to further understand the role of DCs in NEC pathogenesis.

### Neutrophils

Neutrophils are important effector cells of the innate immune system distinguished by their ability to respond rapidly and robustly to tissue trauma, including the intestine. The role of neutrophils in NEC pathogenesis has not been fully elucidated, but there are several studies to note. Small for gestational age (SGA) neonates are more likely to have neutropenia during the first few days after birth, and these infants with neutropenia were at increased risk for developing NEC compared to SGA infants without neutropenia (78). Moreover, in a model of *C. sakazakii*-induced experimental NEC, depletion of neutrophils and macrophages in the lamina propria resulted in increased production of pro-inflammatory cytokines and increased enterocyte apoptosis, thereby exacerbating the disease (79). This suggests both macrophages and neutrophils are important in early infection and their absence intensifies the inflammatory response observed in NEC. One study utilizing TNBS-induced enterocolitis to model NEC found the leukocyte infiltrate relatively devoid of neutrophils in the intestinal tissue (80). Taken together, impairment of neutrophil function is one component of NEC; however, more studies are necessary to determine whether this is a primary observation in NEC or secondary to the disease.

### Peyer’s Patches

Peyer’s patches are unique structures found primarily in the distal intestine that serve as secondary lymphoid tissue. Compared to other secondary lymphoid tissues, Peyer’s patches are distinguished by their significant exposure to a diverse group of antigens, from the gut microbiota to food antigens (81). Antigens are delivered by M cells of the epithelium, and upon activation, generate B cells and plasma cells to maintain mucosal immunity (81). Peyer’s patches appear in the intestine at 11 weeks, but continue to develop throughout gestation (82). Moreover, their numbers increase in proportion to gestational maturation, and thus, premature infants have less Peyer’s patch numbers and decreased maturation (82). It is unknown if this has clinical significance in the setting of NEC. Accordingly, more studies are necessary to determine if Peyer’s patches have a role in NEC as a consequence of prematurity.

## Cell Signaling

The innate immune system of the intestine is intricately involved in the pathogenesis of NEC. An important aspect of the innate immune system is the expression of PRRs, including TLRs and NOD proteins, that can interact with the local gut environment and initiate several signaling pathways. We will review these essential components and their cellular responses with specific attention to aberrant TLR4 signaling, which is well studied in the pathogenesis of NEC.

### PRRs: TLR and NOD

Pattern-recognition receptors are expressed throughout the cells of the body as a means of detecting threats to local homeostasis. The cells of the intestinal epithelium and immune cells express TLRs and NOD proteins, which can detect pathogen-associated molecular patterns such as the LPS of Gram-negative bacteria and flagella and consequently initiate an appropriate response to these bacterial stressors (23).

Specifically, in the case of TLR4 activation, signaling results in nuclear factor-κB (NF-κB) activation, subsequent cytokine production, resulting in an acute inflammatory response (23). TLRs are expressed by intestinal epithelial cells and immune cells of the lamina propria and are involved in epithelial cell proliferation, IgA production, maintenance of tight junctions, and AMP expression (23, 57, 83). TLR4 is of specific interest in the understanding of NEC pathogenesis, as its overexpression in the setting of prematurity yields a significant pro-inflammatory response and dysfunction of the epithelium (84). TLR4 is expressed by intestinal epithelial cells (85), which we will discuss in greater detail with respect to epithelial TLR4 expression and the development of NEC.

However, other TLRs have also been implicated in NEC pathogenesis. For example, TLR2 is expressed throughout the intestinal epithelium, including enterocytes, as well as various immune cells found in the lamina propria. Gram-positive bacteria in the intestine can engage TLR2, which can induce MyD88, which is a major adaptor for TLR2/TLR4, with subsequent NF-κB activation (86). TLR2 through the regulation of tight junctions is able to maintain gut barrier integrity, and thus, its deficiency can predispose the intestine to stress-induced injury (86). The role of TLR2 in NEC has not been fully elucidated thus far. However, in a rat model of NEC, investigators have found upregulation of ileal TLR4 and TLR2 in several studies, which precedes histological evidence of mucosal injury, suggesting a causative role in NEC pathogenesis (87, 88). Moreover, certain therapeutic interventions that reduced NEC severity in animal models downregulated the expression of TLR2 and TLR4, including glutamine (88) and probiotic *Bifidobacterium* (89). Thus, aberrancy in TLR4 signaling is not solely responsible for the pro-inflammatory response observed in NEC.

Moreover, NOD1 is also expressed by intestinal epithelial cells, which in response to the Gram-negative bacterial peptidoglycan, elicits an immune response to induce the formation of gut-associated lymphoid tissue, specifically Peyer’s patches (9, 57). Conversely, NOD2 is expressed highly by monocytes and Paneth cells at baseline, and by enterocytes when under stress (9, 57). NOD2 is important in the cross talk between T-cells and intestinal epithelium to downregulate inflammation by inhibiting TLR signaling (9, 57). One study demonstrated that NOD2 activation inhibited TLR4 in enterocytes, thereby decreasing enterocyte apoptosis and attenuating the severity of experimental NEC (90). In this study, NOD2 provided protection from TLR4-mediated enterocyte apoptosis *via* a novel SMAC-diablo pathway (90). This suggests that modulation of this cross talk may provide a potential means of decreasing NEC severity (83, 90). Furthermore, NOD2 is important in regulating commensal bacteria such that NOD2-deficient mice had decreased ability to prevent colonization by pathogenic bacteria in the intestine (91). Conversely, germ-free mice had significantly decreased NOD2 expression, but this expression was inducible with the introduction of commensal bacteria, demonstrating the feedback mechanism by which NOD2 regulates the gut microbiota (91). Upon ligand binding, both NOD1 and NOD2 activate NF-κB, and independently function by activating this signaling pathway, but can also modulate TLR4 signaling in this way (92). Thus, TLR and NOD signaling exemplify the delicate interaction between luminal bacteria and the innate immune system of the gut and how perturbations in these pathways may lead to NEC development.

### TLR4 Signaling

One of the major cornerstones in understanding the development of NEC is the role that TLR4 signaling plays in the pathogenesis (59, 85, 93–100). Activation of TLR4 within the intestinal epithelium in the setting of prematurity results in decreased enterocyte proliferation, increased enterocyte apoptosis, disruption of intestinal barrier integrity, and bacterial translocation, resulting in a systemic inflammatory response (26, 84, 95, 97). As a result of bacterial translocation, TLR4 is activated on the endothelium of premature gut, leading to impaired blood flow and subsequent intestinal ischemia *via* reduction of endothelial nitric oxide synthase (eNOS) (100, 101). The differential response to stress between premature and full term neonates rests in the increased expression of TLR4 in prematurity (96). TLR4 is expressed at high levels in the developing intestine as it is involved in normal gut development in both mice and humans (8). In the setting of prematurity, TLR4 expression remains elevated, resulting in a hyper-active, exaggerated response to stressors upon colonization by bacteria (8).

Furthermore, TLR4 is necessary for the development of NEC, and its enhanced expression is not a consequence of the disease. In studies utilizing TLR4-mutant mice strains (C3H/HeJ mice), lack of functional TLR4 was protective against the development of NEC, such that wild-type mice had increased NEC severity, increased enterocyte apoptosis, reduced enterocyte proliferation, and impaired restitution compared to TLR4-mutant mice (97). These results were further supported utilizing mice with either global TLR4 deletion or intestinal-specific TLR4 deletion (26). Both groups of mice were protected from the development of experimental NEC with preservation of mucosal integrity and minimal elevation of pro-inflammatory cytokines (26). These studies highlight the causative role of aberrant TLR4 signaling expressed by IECs in NEC pathogenesis.

Aberrant TLR4 signaling also has a direct role in the breakdown of the gut barrier in NEC. In healthy mucosa, healing of the epithelium occurs in two phases, intestinal restitution followed by enterocyte proliferation, whereby healthy IECs migrate to injured mucosa followed by increased generation of IECs from stem cells of the intestinal crypts (18). TLR4 signaling impairs IEC migration, thus impairing restitution (102, 103). Moreover, enterocyte proliferation is significantly decreased in NEC, diminishing the ability to heal in the setting of mucosal injury (18, 95, 97–99). Importantly, autophagy is a response to cellular stress and has been found to be upregulated in NEC (104). Furthermore, TLR4 signaling induces autophagy of enterocytes in both mouse and human studies (105). An experiment utilizing intestinal epithelial-specific autophagy gene ATG7 conditional knockout mice demonstrated autophagy *via* ATG7 was required for NEC development as these mice were protected from NEC (105). TLR4-induced autophagy leads to impaired enterocyte migration, demonstrating the effects of TLR4 signaling on the epithelium are multifaceted and interconnected (105). Ultimately, the deficiency in mucosal repair *via* enterocyte restitution and proliferation in the setting of NEC weakens the integrity of gut, allowing for bacterial translocation and the downstream inflammatory response observed in this disease.

### Genetic Risk Factors

The study of genetic risk factors in NEC is an important component in understanding the signaling pathways that involve the innate immune system. There are several genetic association studies evaluating the relationship between specific genetic risk factors and the development of NEC (106). Genetic variation that affects TLR signaling increases the predisposition of the premature intestine to inflammatory aberrancy. The gene *SIGIRR* is important in the inhibition of LPS-induced inflammation (107). Loss-of-function mutations of *SIGIRR* result in unregulated TLR signaling, thereby predisposing infants to NEC (107). One study specifically evaluated the roles of single nucleotide polymorphisms of important genes in TLR signaling. They found in studying the blood samples of very low birth weight infants a relationship between *NFKB1* and *NFKBIA* variants and the development of NEC, such that *NFKB1* increased susceptibility to NEC, whereas *NFKBIA* decreased susceptibility to NEC (108). However, there was no association between *TLR2, TLR4, TLR5, TLR9, IRAK1*, and *TIRAP* genes and the development of NEC in this patient population (108). Genetic risk factors for NEC have not just been limited to TLR and NOD signaling pathways. For example, one study evaluating the autophagy gene ATG16L1 in premature infants found that hypomorphic variants conferred protection for the development of NEC (109). This study highlights the breadth of possible genetic risk factors in the NEC. Taken together, genetic predisposition to NEC is valid, and further large scale studies will likely reveal more genetic relationships and disease development.

## Gut Microbiota

The intricate relationship between gut microbiota and mucosal immunity is central to the discussion of NEC pathogenesis. The gut is exposed to a multitude of microbes, and it is the role of the intestinal immune system to distinguish commensal bacteria from pathogenic bacteria, particularly as the gut flora develops early in life. There are several variables that influence the microbial composition of the intestine; however, new evidence suggests that disruption of normal bacterial flora is involved in NEC pathogenesis. We will now explore the role of gut microbiota in the setting of prematurity.

### Factors Influencing Gut Microbiota

There is a significant interest in understanding the composition of the gut microbiota and its relationship to the pathogenesis of NEC. Several factors influence the neonatal microbiota, including gestational age, mode of delivery (vaginal vs. cesarean section), antibiotic treatment, and diet (breast milk vs. formula feedings) (110). Colonization occurs in two waves, and the first wave is dependent on the mode of delivery (111). For example, one study found that compared to infants born vaginally, infants born *via* cesarean section had decreased populations of *Bifidobacteria* and *Bacteroides*, while there was an increased population of *Clostridium difficile* (112). The second wave is dependent on feeding method, which often differs between premature and term infants, as breast milk and formula feedings have different bacterial compositions and access to breast milk can be limited in prematurity with delayed initiation of enteral feeding in preterm newborns (113). More specifically, formula-fed infants have increased populations of *Enterobacteriaceae, Bacteroides* species, and *C. difficile* in the stool compared to infants fed breast milk (112, 114).

Moreover, duration of antibiotic exposure is associated with the development of NEC (115, 116). In one retrospective study investigating the association between antibiotic exposure and subsequent diagnosis of NEC in infants, they found that antibiotic exposure duration is associated with increased risk of developing NEC (115). When sepsis was eliminated as a potential confounder, the probability of developing NEC was increased 20% per day of antibiotic exposure (115). Strikingly, antibiotic exposure greater than 10 days in neonates resulted in an approximately threefold increased risk of developing NEC (115). Another retrospective study aiming to assess the association between initial antibiotic therapy in extremely low birth weight infants and NEC, found there was an increased risk of developing NEC after initial empiric antibiotic treatment in the first three postnatal days (116). Empiric antibiotic treatment for greater than 5 days, which the study defined as prolonged antibiotic treatment, was associated with the development of NEC and death in extremely low birth weight infants in the setting of sterile blood cultures (116). These studies highlight the need to be judicious in the implementation of empiric antibiotic treatment in preterm infants.

A recent study utilizing 16S rRNA gene pyrosequencing suggests the most important variable influencing the composition of premature gut microbiota is the degree of prematurity (117). Using 922 specimens from 58 subjects, they found an ordered, tightly controlled microbial progression of bacterial classes: Bacilli to Gammaproteobacteria to Clostridia (117). Other factors, including antibiotics, mode of delivery, and age influenced the pace of the progression of bacterial classes, but the particular sequence of development remained the same (117). This is important as more studies demonstrate the significance of dysbiosis and the development of NEC (113, 118–122).

### Dysbiosis in NEC

The differences in microbial colonization between preterm infants and term infants suggests the possible role of dysbiosis on the pathogenesis of NEC. The onset of NEC occurs 2–6 weeks of life with the highest risk of NEC occurring at a corrected age of 29–33 weeks (123), only after microbial colonization of the gut (2, 124, 125), highlighting the role of gut microbiota in disease pathogenesis. To date, no specific microbial pathogen has been identified to be responsible for the development of NEC (119, 124, 125). However, resected intestinal tissue with active NEC demonstrated increased microbial burden and an abundance of strict anaerobes with a decrease in community diversity (126). Moreover, until recently, it was unclear if abnormal gut microbiota is a cause or consequence of NEC (127). A prospective case–control study by Warner et al. (127) helps to define the role that the microbiota plays in NEC development. In 166 very low birth weight infants, 3,586 stool samples were prospectively collected, and of these subjects, 46 developed NEC (127). Differences between the NEC cases and matched controls emerged after 1 month with a predominance of Gammaproteobacteria and decreased quantities of Negativicutes and Clostridia in infants that went on to develop NEC, with the strongest correlation between bacteria composition and development of NEC occurring prior to 27 weeks gestation (127). This study suggests an increased concentration of Gram-negative facultative bacteria is detrimental, possibly due to their ability to activate TLR4 *via* LPS (84, 122). Conversely, infants with NEC had significantly decreased obligate anaerobes (127), which interestingly produce anti-inflammatory SCFAs (128). These results suggest that interventions focused on modulation of the gut bacteria may play a role in preventing NEC.

## Protective Strategies Against NEC

**Figure 2:**
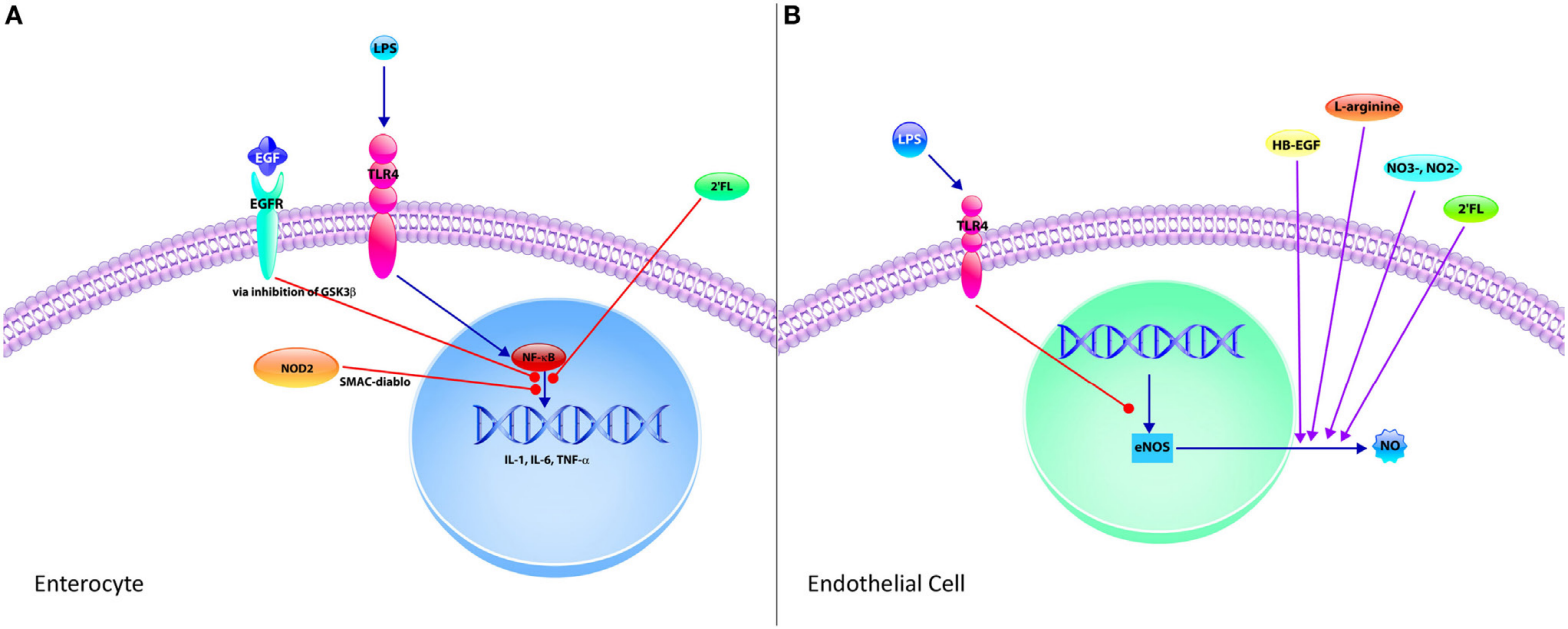
**Mechanisms of protective strategies in necrotizing enterocolitis (NEC)**. **(A)** The enterocyte is the predominant cell type of the epithelium and significantly contributes to the exaggerated pro-inflammatory response observed in NEC upon activation of toll-like receptor 4 (TLR4) by Gram-negative bacteria lipopolysaccharide (LPS). TLR4-mediated activation of nuclear factor-κB (NF-κB) results in increased expression of pro-inflammatory cytokines, such as IL-1, IL-6, and TNF-α. However, this signaling pathway can be attenuated through various mechanisms. Epidermal growth factor (EGF), which is found in breast milk and amniotic fluid, inhibits this pathway *via* inhibition of glycogen synthase kinase-3 beta (GSK3β). The human milk oligosaccharide 2′-fucosyllactose (2′FL) also attenuates the TLR4 pro-inflammatory signaling pathway. Finally, intracellular pattern recognition receptor nucleotide-binding oligomerization domain-containing protein 2 upon stimulation by pathogen associated molecular patterns *via* second mitochondria-derived activator of caspase/direct inhibitor of apoptosis-binding protein with low pI (SMAC-diablo). **(B)** Endothelial cells have an integral role in the pathogenesis of NEC. Endothelial cells express TLR4, and upon stimulation by LPS following gut barrier dysfunction, decrease the expression of endothelial nitric oxide synthase (eNOS), thereby reducing the formation of nitric oxide (NO), an important vasodilator. This results in intestinal ischemia and subsequent NEC. However, several protective strategies increase the production of NO, including heparin-binding EGF-like growth factor (HB-EGF) and 2′FL by increasing expression of eNOS. Nitrates $\left({\text{NO}}_{3}^{-}\right)$ and nitrites $\left({\text{NO}}_{2}^{-}\right)$ can be found in breast milk and are precursors to NO, thus increasing NO production. Finally, l-arginine is required for NOS-dependent formation of NO. Supplementation with HB-EGF, nitrates, nitrites, and l-arginine are protective in NEC with their ability to improve intestinal blood supply *via* NO-mediated vasodilation.

With advancements in our knowledge of NEC, there are several potential preventative and therapeutic strategies that did not exist decades ago. We will review several of these strategies with respect to how they influence the intestinal immune system (Figure 2). These advancements target several aspects of the intestinal immune system, such as TLR4 signaling modulation, gut barrier integrity, immune cell composition, and the gut microbiota, all of which have been described with their individual roles in NEC. There have been several studies that demonstrate the importance of providing breast milk to premature infants instead of formula. Human breast milk contains several bioactive components beneficial to the neonate some of which include: epidermal growth factor (EGF), heparin-binding EGF-like growth factor, platelet-activating factor (PAF) acetylhydrolase, human milk oligosaccharides (HMOs), nitrates/nitrites, l-arginine, lactoferrin, and probiotics (8, 129, 130). We will review several breast milk components and the evidence supporting the mechanisms by which they impact the intestinal mucosal immunity during NEC. We will also discuss the use of antenatal corticosteroids and delayed umbilical cord clamping in the prevention of NEC.

### Breast Milk

Breast milk has a well-established role in the prevention of NEC and clinically represents one of the most effective strategies in decreasing the incidence and progression of NEC (130). New pre-clinical evidence has demonstrated that breast milk is capable of attenuating the TLR4-mediated pro-inflammatory response integral to NEC pathogenesis by activating the receptor for epidermal growth factor (EGFR), revealing the interplay between the EGF pathway and the hallmark TLR4 signaling of NEC (95). These inhibitory effects on TLR4 signaling were mediated by breast milk preventing the activity of the downstream target glycogen synthase kinase 3β, resulting in enhanced mucosal healing, intestinal stem cell proliferation and decreased enterocyte apoptosis (95). In seeking to determine the particular component in breast milk that mediated the protective effects, when EGF was either removed or its receptor, EGFR was inhibited, the protection on experimental NEC and TLR4 signaling was abolished (95). Taken together, these mechanistic studies describe one of the ways breast milk protects the intestinal epithelium against NEC.

### Epidermal Growth Factor

Several other studies have looked at the protective role of EGF itself against NEC. In a neonatal rat model of NEC, administration of EGF alone reduced both the incidence and severity of the disease (131). EGF administration resulted in significantly decreased epithelial permeability, normalized expression of tight junction proteins, increased goblet cells (28), and inhibited enterocyte apoptosis (132), all of which are protective hallmarks in NEC (26, 59, 95). Moreover, another mechanism by which EGF provides protection against NEC is inhibition of autophagy (133), a lysosomal pathway of self-digestion that has been shown to be activated in mice and infants with NEC (105). Additionally, a recent study demonstrates that a hypomorphic variant in an autophagy-related gene, ATG16L1 is associated with NEC in premature infants (108). This further underlies the importance of determining which infants are the most susceptible to NEC, so that protective strategies including EGF administration may be tailored to those individuals.

### Amniotic Fluid

As discussed above, EGF administration mediates several protective effects on the intestine and is found in breast milk as well as amniotic fluid. We demonstrated that amniotic fluid inhibited TLR4-mediated inflammatory signaling in the fetal and neonatal intestinal epithelium in a manner dependent on EGFR (94). Intestinal EGFR expression was low in premature infants with NEC compared to that of a fetus or at the time of reanastamosis after NEC had resolved (94). The protective effects of amniotic fluid have been described in experimental NEC models in several species including neonatal mice (94), rats (134), and piglets (135). Other investigators have examined amniotic fluid outside the setting of EGF in the study of NEC. In a neonatal rat model of NEC, administration of amniotic fluid stem cells attenuated NEC by increasing enterocyte proliferation and decreasing apoptosis in a cyclooxygenase 2 dependent mechanism (136). In other studies, the administration of amniotic fluid resulted in differences in gut microbiota and reduced intestinal permeability compared to controls (137). Taken together, the ingredients in both breast milk and amniotic fluid provide important protective mechanisms to counter-regulate the detrimental pro-inflammatory effects in the intestine afflicted by NEC.

### Heparin-Binding EGF-Like Growth Factor (HB-EGF)

Heparin-binding EGF-like growth factor is found in breast milk and amniotic fluid and provide numerous protective effects in models of intestinal injury and NEC (138). In mice, overexpression of the HB-EGF gene is protective against the development of NEC (139), whereas deletion of HB-EGF increases susceptibility to experimental NEC (140). As described previously, an important component in the pathogenesis of NEC is the disruption of gut perfusion and decreased expression of the vasodilatory molecule eNOS (100). In neonatal mice subjected to experimental NEC, impairment of intestinal microvascular blood flow was improved, and there was decreased epithelial injury with administration of HB-EGF (141). Moreover, HB-EGF promotes angiogenesis with upregulation of eNOS and subsequent production of nitric oxide *via* the PI3K pathway (142, 143).

Heparin-binding EGF-like growth factor is also unique in its ability to modulate the intestinal immune system. For example, there are subtypes of macrophages, which can either be pro-inflammatory (M1) or anti-inflammatory (M2) (144). Macrophage infiltration in NEC was marked by a predominance of M1 pro-inflammatory macrophages and treatment with HB-EGF resulted in increased M2 anti-inflammatory macrophages, thereby protecting against experimental NEC. Moreover, HB-EGF also helps in maintaining gut barrier integrity with increased enterocyte proliferation (145) and migration (146) as well as decreased apoptosis of enterocytes (147), which is a common theme among the potential protective strategies in NEC.

### Platelet-Activating Factor Acetylhydrolase (PAF-AH)

Platelet-activating factor is a potent phospholipid inflammatory mediator involved in the pathogenesis of NEC in human and animal studies (148, 149). Activation results in epithelial cell damage, increased apoptosis, increased mucosal permeability, disruption of tight junctions, leukocyte and platelet aggregation, and vasoconstriction, disrupting mucosal integrity (148, 149). Importantly, PAF-AH is capable of catabolizing PAF and decreases the destructive properties of PAF in the intestine (150). Additionally, breast milk contains PAF-AH, which is one component in breast milk thought to provide protection against the development of NEC (150, 151). The clinical relevance of PAF is demonstrated by the findings that compared to controls, infants with NEC display increased plasma and stool PAF concentrations and decreased plasma PAF-AH, which is responsible for PAF breakdown (152–154). In neonatal mouse and rat models, NEC susceptibility decreased with inhibition of PAF and increased with PAF-AH depletion (155–157). Of note, PAF was found to induce TLR expression in the intestine, providing a connection between two pathways involved in NEC pathogenesis, thereby suggesting luminal PAF in the preterm intestine may upregulate TLR expression and subsequently promote a pro-inflammatory state (158). Taken together, PAF-AH may serve as an important target by inhibiting intestinal inflammation and epithelial disruption.

### Human Milk Oligosaccharides

Human milk oligosaccharides are carbohydrates recently heralded as protective components within breast milk. There are several means by which HMOs are protective in the setting of NEC. We recently demonstrated that 2′-fucosyllactose (2′FL) is an abundant HMO that is protective against NEC in mice *via* modulation of the vasodilatory molecule, eNOS expression and subsequently enhancing intestinal perfusion (101), which has been previously shown to be impaired in NEC (100). Moreover, 2′FL is capable of attenuating inflammation, and we demonstrated that 2′FL decreased the expression of several pro-inflammatory markers including IL-6, IL-1β, inducible nitric oxide synthase, and TLR4 (101). Importantly, HMOs have been shown to influence bacterial colonization in the intestine during the critical neonatal period in several studies (159–165). In another study, the HMO 2′FL inhibited the release of IL-8 by IECs in the setting of bacterial infection by attenuating the expression of CD14 (166). However, this effect was not observed in IECs that were not exposed to the bacterial pathogen, showing that HMOs are capable of modulating specific inflammatory pathways during infection (166). Another HMO, disialyllacto-N-tetraose, was capable of reducing the NEC severity in neonatal rats (167). Taken together, these studies advance our understanding of the effects of HMOs in preventing against NEC and may provide a nutritional preventative strategy worth pursuing.

The breast milk composition of HMOs varies among mothers, which in turn influences the microbiota of their offspring. Specifically, mothers with inactive alleles of the gene *fucosyltransferase 2* (FUT2) are referred to as non-secretor mothers (168), and this mutation leads to changes of the microbiota of their infants. Specifically, infants in this setting had delayed establishment of the genus *Bifidobacterium*, which are early colonizers in breastfed infants (168). This suggests HMOs can enrich certain beneficial populations of bacteria. Moreover, since the individual composition of breast milk varies widely, attention to these details may provide a novel strategy of personalized breast milk fortification to prevent NEC in preterm infants.

### Nitric Oxide

One of the hallmarks of NEC pathogenesis is the disruption of the microcirculatory perfusion to the gut, which is regulated by endothelial expression of TLR4 (100). Specifically, endothelial TLR4 signaling impairs intestinal perfusion and decreases eNOS expression and accordingly nitric oxide (NO), such that mice with selective endothelial TLR4 deletion exhibited preserved mucosal integrity and decreased intestinal ischemia (100). In this context, TLR4 signaling promotes bacterial translocation with the disruption of the epithelial barrier, intensified by impaired intestinal perfusion by decreased expression of eNOS with the activation of endothelial TLR4.

Human breast milk contains sodium nitrate, which is a precursor to nitrite and NO production, a key vasodilatory molecule involved in maintaining intestinal perfusion (100). Interestingly, the concentration of sodium nitrate is higher in breast milk as compared to formula (100), and when compared to adults, infants in the NICU receive significantly less dietary nitrites and nitrates (169). Bacterial conversion of nitrates to nitrites first occurs in saliva, which can then be converted to nitric oxide *via* both NOS-independent mechanisms in the stomach and NOS-dependent mechanisms in the intestine from the amino acid l-arginine (170). This illustrates the interaction of bacteria and the gastrointestinal system and also highlights the importance of having dietary nitrates and nitrites in the form of breast milk during the critical neonatal period. This further suggests there is the opportunity to improve the deficits in dietary nitrites and nitrates inherent to neonates to prevent NEC. Moreover, studies have shown that enteral supplementation of l-arginine was not only safe but was able to reduce incidence and severity of NEC in very low birth weight preterm infants (171, 172). Accordingly, l-arginine, nitrates, and nitrites in the neonatal period are not only important but given their ability to modulate the microcirculation of the preterm intestine, they have utility as a preventative strategy for NEC.

### Lactoferrin

Lactoferrin is found in secretory fluids including breast milk and colostrum, where it acts an important member of mucosal immunity with its antimicrobial properties (173), ability to modulate the gut microbiota (174), and maturation of the intestine by inducing enterocyte growth and proliferation (175). Accordingly, the use of lactoferrin supplementation in preterm infants to prevent complications has been studied. The most recent Cochrane Review suggests that there is utility in the administration of oral lactoferrin prophylaxis due to the decrease in the development of NEC and late-onset sepsis without adverse effects (176–180). Another study evaluated the use of recombinant human lactoferrin (talactoferrin or TLf) in infants with a birth weight of 750–1,500 mg as means of reducing infection (181). There was no associated toxicity in the administration of TLf, and there was a trend toward less infectious morbidity in infants treated with TLf (181). Thus, lactoferrin administration in low birth weight infants is a promising preventative strategy against NEC with no adverse effects reported thus far.

### Probiotics

Probiotics have generated significant interest in the prevention of NEC given the increasing data regarding the relationship between the gut microbiota and disease development. Prophylactic enteral probiotics show significant promise in the prevention of NEC. A Cochrane Review concluded probiotics in the setting of prematurity prevent severe NEC and decrease all-cause mortality (182). A more recent systematic review and meta-analysis utilizing additional studies supported these findings (183). As probiotics continue to be investigated, it is important to recognize there is significant heterogeneity with organisms chosen and dosing regimens across the studies. *Bifidobacterium lactis* has been studied in isolation in clinical studies. In a prospective randomized case–control study, administration of *B. lactis*-supplemented formula resulted in decreased intestinal permeability as measured by a sugar absorption test, suggesting *B. lactis* is important in maintaining epithelial integrity (184). In another clinical study, *B. lactis* Bb12 supplementation influenced the gut microbiota such that supplementation increased the number of *Bifidobacterium* spp. and reduced the number of *Enterobacteriaceae* and *Clostridium* spp., which may be implicated in NEC pathogenesis, in stool samples of preterm infants (185). It is important to note that the largest clinical trial for a specific probiotic, *Bifidobacterium breve* BBG-001, found no benefit in the administration of this probiotic in preterm infants to prevent NEC (186). More clinical studies evaluating the utility of specific organisms within probiotic regimens are necessary.

Moreover, there are several mechanisms by which probiotics can prevent the development of NEC (187). Two studies highlight our present knowledge of NEC pathogenesis as related to mucosal immunology. *Bifidobacterium adolescentis* is able to alter TLR4, TOLLIP, and SIGIRR expression in preterm neonatal rats, suggesting it is able to downregulate the TLR4-mediated pro-inflammatory response observed in NEC (188). Oral administration of *Lactobacillus rhamnosus* HN001 attenuated NEC severity in both premature piglets and newborn mice and the mechanism mediating this effect was *via* TLR9 activation (189). *Lactobacillus reuteri* in neonatal mice was able to increase enterocyte migration, enterocyte proliferation, and crypt height of the epithelium. This counters the impairment in enterocyte migration and proliferation that serve as hallmarks of NEC-mediated gut barrier dysfunction (190). These studies suggest probiotics are able to interact with the mucosal immunity of the gastrointestinal tract, thereby offering a viable preventative strategy in clinical NEC management.

### Antenatal Steroids

Corticosteroids are often administered in the antenatal period to reduce mortality and morbidities associated with prematurity. Based on a Cochrane Review, the use of a single course of antenatal corticosteroids is recommended for all women at risk for preterm birth (191). In addition to accelerating fetal lung maturation, antenatal corticosteroids are associated with decreased likelihood for the development of necrotizing enterocolitis analyzing 8 studies with 1,675 infants total (191). A more recent clinical study supports these findings, in which there were significant differences between infants that received full course antenatal steroids and infants that did not, such that prompt administration of a full course of antenatal corticosteroids decreased the incidence of NEC and mortality (192). Presently, it is unknown the mechanism by which corticosteroids are protective in NEC. However, with our knowledge of the primary role of immune cells in the pro-inflammatory response in NEC, corticosteroids serve as an interest of study with their ability to modulate the immune system.

### Umbilical Cord Clamping

There is debate on the optimal interval of time between infant delivery and umbilical cord clamping in the setting of prematurity. Delayed cord clamping provides more placental transfusion between the placenta and the newborn, whereas immediate cord clamping allows for immediate resuscitation of the premature newborn by a neonatologist (193). A Cochrane Review found that delayed cord clamping decreased the incidence of NEC (193); however, larger studies are necessary to evaluate this finding as well as the long-term effects of immediate vs. delayed cord clamping. In a more recent study, umbilical cord milking, which utilizes the same principle behind delayed umbilical cord milking also conferred protection against the development of NEC in preterm infants (194). The mechanisms by which umbilical cord clamping and umbilical cord milking provide protection against NEC are incompletely understood and warrant further investigation.

### Immunoglobulins

The roles of B cells and immunoglobulins in the setting of NEC are not fully understood in the context of disease development and preventative strategies. A trial of oral immunoglobulins administration has been tried for their presumed immunoprotective effects as prophylaxis in preterm infants (195). However, based on a recent Cochrane Review of three randomized trials, there is no protective role against NEC with the oral administration of immunoglobulins in the neonatal period, specifically with IgG or a combination of IgG/IgA, as these studies did not demonstrate a significant reduction in NEC incidence (195–198). A trial of oral IgA alone powered for the prevention of NEC in low birth weight neonates would be beneficial given the known beneficial properties of sIgA in breast milk (195). Nonetheless, more studies are necessary to appreciate the role of IgA in NEC pathogenesis and as a viable prophylactic or treatment strategy.

## Conclusion

Necrotizing enterocolitis is a devastating disease of prematurity and recent evidence demonstrates incredible promise for prevention with continuous advancements in the field. These advancements are not limited to mucosal immunology as recent attention to the interplay of intestinal immune system, which includes epithelial integrity, immune cell composition, TLR4 signaling, intestinal microbiota, has yielded expansion of our understanding of the disease and how to best prevent its development. One of the most important themes of this review is the dynamic interaction of the components that comprise the mucosal immunity of the intestine. Several aspects are integral to maintain intestinal homeostasis, and there is no one component singularly responsible for the development of NEC. Rather, it is an intricate balance of many variables, which yield numerous therapeutic potentials in treating and preventing this disease. The goal of this review was to highlight these recent innovations and mechanistic insights into how administration of various treatments impacts the intestinal immune system and may alleviate NEC in premature infants.

## Author Contributions

All authors listed have made substantial, direct, and intellectual contribution to the work and approved it for publication.

## Conflict of Interest Statement

The authors declare that the research was conducted in the absence of any commercial or financial relationships that could be construed as a potential conflict of interest.
